# Etonogestrel Subdermal Implant in Adolescents: Everything We Should Know to Conduct Proper Counseling, a Narrative Review

**DOI:** 10.3390/clinpract15020027

**Published:** 2025-01-27

**Authors:** Alessandro Messina, Safae Elmotarajji, Eleonora Dalmasso, Costanza Valentini, Valentino Remorgida, Livio Leo, Alessandro Libretti, Bianca Masturzo

**Affiliations:** 1 Department of Obstetrics and Gynecology, University Hospital “Degli Infermi”, 13875 Ponderano, Italy; alessandro.messina@aslbi.piemonte.it (A.M.); safae.elmotarajji@aslbi.piemonte.it (S.E.); eleonora.dalmasso@unito.it (E.D.); costanza.valentini@unito.it (C.V.); bianca.masturzo@aslbi.piemonte.it (B.M.); 2 Health Science Interdisciplinary Center, Scuola Superiore Sant’Anna, 56126 Pisa, Italy; 3 Department of Gynaecology and Obstetrics, University Hospital Maggiore della Carità, 28100 Novara, Italy; valentino.remorgida@uniupo.it; 4 Department of Obstetrics and Gynecology, Hospital Beauregard, AUSL Valleè d’Aoste, 11100 Aosta, Italy; lleo@ausl.vda.it

**Keywords:** etonogestrel, subdermal implant, contraception, adolescents

## Abstract

**Background/Objectives**: Comprehensive sexual education and access to contraceptives play a vital role in alleviating the economic, health, and social challenges associated with unplanned pregnancies in adolescents. According to the World Health Organization (WHO), adolescence refers to the transitional stage from childhood to adulthood, encompassing individuals aged 10 to 19. This period is critical for reproductive decision making, making it essential to closely observe patterns of sexual activity and contraceptive use among young people. Despite advancements, many adolescents still encounter significant barriers to accessing and effectively utilizing contraceptive methods. **Methods**: A bibliographic search was performed across three major biomedical databases: PubMed, Embase, and CINAHL. The research question was developed utilizing the PIO (Population, Intervention, Outcome) framework to guide the investigation. **Results**: Long-acting reversible contraceptives (LARCs), including the etonogestrel (ENG) implant, have been recognized for their ability to significantly reduce maternal and neonatal mortality and morbidity as well as decrease the incidence of unsafe abortions. **Conclusions**: Access to adequate counseling and effective contraceptive services can profoundly impact young people’s lives, preventing unplanned pregnancies and promoting optimal sexual and reproductive health.

## 1. Introduction

Sexual education and access to contraceptives are crucial in reducing the economic, health, and social burdens of unplanned pregnancies among adolescents [1,2]. Adolescence, defined by the World Health Organization (WHO) as the transition period between childhood and adulthood, spans from ages 10 to 19 [1]. Given the significance of reproductive decisions made during this phase, it is essential to closely monitor sexual activity and contraceptive use among young people. However, despite progress, adolescents continue to face considerable challenges in accessing and effectively using contraceptive methods.

Unplanned pregnancies among adolescents have significant economic and social consequences for society as a whole, according to a research of the WHO [2]. Additionally, a study conducted in the USA estimated that approximately 3.9 million unsafe abortions occur annually among girls aged 15 to 19, contributing substantially to maternal mortality and morbidity as well as long-term health issues [3]. In 2019, 55% of unplanned pregnancies among adolescents ended in abortion in an Indian cohort [4]. Contraceptive use and sexual behavior are key determinants in the rates of unplanned pregnancies among youth. A high percentage of adolescents reported using a contraceptive method during their first sexual encounter, with rates of 79% among those who initiated sexual activity between ages 15 and 16 and 83% among those aged 17 to 19, according to a U.K.-based population [5].

However, between 2017 and 2019, only 22.1% of sexually active adolescents used a highly or moderately effective contraceptive method during their most recent sexual encounter, well below the target of 36.8% [6]. The target of 36.8% refers to the Healthy People 2020 objective set by the U.S. Department of Health and Human Services (HHS). This objective aimed to increase the proportion of sexually active adolescents who use highly or moderately effective contraceptive methods during their most recent sexual encounter. The target was established as part of a broader initiative to promote evidence-based strategies for improving adolescent health outcomes and reducing unintended pregnancies in this age group. The specified target percentage (36.8%) represents a benchmark for national improvement in contraceptive use among adolescents [6]. These data indicate a need for further efforts to improve the adoption of effective contraceptive methods among youth. The reduction in unplanned pregnancies among adolescents has been largely attributed to increased use of contraceptives, with a notable rise in the adoption of long-acting reversible contraceptives (LARCs), such as the etonogestrel subcutaneous implant (ENG). LARCs, including the ENG implant, have been recognized for their ability to significantly reduce maternal and neonatal mortality and morbidity as well as decrease the incidence of unsafe abortions [7]. The main categories of LARCs include [7] intrauterine devices (IUDs):

- Copper IUD: a non-hormonal device that prevents pregnancy by creating an environment toxic to sperm;
- Hormonal IUD: releases progestin (levonorgestrel) to thicken cervical mucus, inhibit sperm movement, and alter the uterine lining;
- Contraceptive implants: a small rod placed under the skin of the upper arm that releases progestin to prevent ovulation and thicken cervical mucus.

From 2000 to 2020, the use of modern contraceptive methods increased from 663 million to 851 million users, with a projected additional 70 million users by 2030 [8]. During the same period, LARC adoption among adolescents grew from 0.4% to 19.2%, with the use of the ENG implant rising from 0.1% to 13.3% [9]. Despite these encouraging statistics on LARC adoption, the rates of unplanned pregnancies among adolescents remain high, indicating that significant challenges persist in ensuring equitable access and effective use of ENG implants among this population.

LARCs are particularly effective in preventing unintended pregnancies among adolescents, being 40 times more effective than oral contraceptives, as their efficacy does not depend on user adherence [7]. The American College of Obstetrics and Gynecology (ACOG), the American Academy of Pediatrics (AAP), the Centers for Disease Control and Prevention (CDC), and the Society of Family Planning recommend LARCs, including etonogestrel implants, as first-line contraceptive options for adolescents [10,11,12,13]. These methods, often referred to as “forgettable contraceptives”, provide an option that requires no ongoing intervention after insertion and maintains its effectiveness for years, making them ideal for young people who may struggle with daily contraceptive routines [10,11].

The existing literature on the use of contraceptive implants among adolescents highlights good acceptability and continuity rates similar to those observed in adults [10]. The United Nations 2030 Agenda emphasizes the importance of ensuring universal access to sexual and reproductive health services, including family planning, information, and education, with the integration of reproductive health into national strategies and programs (goal 3.7) [14]. This ambitious goal reflects the need for ongoing commitment to improving access to reproductive health services and reducing disparities.

Despite this regulatory framework, adolescents often hesitate to seek healthcare for contraception due to concerns about pelvic exams, cultural taboos, prevalent biases, and fears about side effects. These factors can lead to improper use of contraceptives and, consequently, reduced effectiveness. ACOG and AAP guidelines recommend a reproductive health visit between ages 13 and 15, aimed at establishing trust with the healthcare provider and discussing the most appropriate contraceptive options. It is crucial that during contraceptive counseling, adolescents are empowered to make informed decisions free from external pressures. Addressing specific barriers, such as confidentiality, cost, and concerns about pelvic exams, is essential to improving access to contraceptives [15].

Access to adequate counseling and effective contraceptive services can profoundly impact young people’s lives, preventing unplanned pregnancies and promoting optimal sexual and reproductive health [16]. This review aims to identify barriers and opportunities to optimize contraceptive counseling and improve access and use of ENG implants among adolescents.

## 2. Materials and Methods

To explore the topic effectively, a bibliographic search was carried out across three biomedical databases: PubMed, Embase, and CINAHL. The search strategy was broad, applying no time limits to maximize inclusivity and relevance to the study’s scope. The research question was constructed using the PIO framework, adapted for this review:

- P (Population): adolescent individuals considering or using the etonogestrel subdermal implant;
- I (Intervention): counseling regarding the use and management of the implant;
- O (Outcome): improved understanding, informed decision making, and optimized implant use.

The inclusion and exclusion criteria, along with the grouping, search strategy, and methods for data synthesis in this review, are detailed below. This is in agreement with PRISMA guidelines (see Supplemental Material for PRISMA checklist and flow chart; registration not required due to the narrative synthesis of the evidence).

Inclusion and Exclusion Criteria

Inclusion criteria included the following:

- Studies focusing on adolescents (10–19 years) considering or using the etonogestrel subdermal implant;
- Reports addressing counseling regarding the implant, its use, and its outcomes;
- Data on improved understanding, informed decision making, and optimized use of the implant.

Exclusion criteria included the following:

- Studies involving the wrong population (e.g., non-adolescents);
- Reports not within the scope of the research focus;
- Studies not reporting outcomes of interest or lacking specified adolescent data;
- Reports focusing on abortion methods unrelated to the ENG implant.

Grouping for Syntheses

Studies included in the review were grouped based on the following:

- The outcomes related to contraceptive use and counseling effectiveness;
- Factors affecting adoption, retention, or discontinuation of the ENG implant.

Sources Searched

The sources searched included the following:

- Databases: PubMed, Embase, and CINAHL;
- Other Sources: reference lists of relevant articles;
- No time limits were applied to the search for inclusivity;
- Last Search Date: January 2025.

A PIO (Population, Intervention, Outcome) framework was used:

- Population: adolescents considering or using the ENG implant;
- Intervention: counseling and implant use;
- Outcome: understanding, decision making, and implant usage optimization.

No specific filters or limits were mentioned, ensuring a comprehensive search.

Study Screening and Data Collection

The following methodology was utilized:

- Screening: duplicate records (450) and irrelevant studies (120) were excluded before screening 2330 records. The process led to the inclusion of 17 studies;
- Reviewers: the document does not specify the number of reviewers or whether the process was independent. This should be clarified in the final report;
- Automation Tools: none applicable.

Outcome and Variable Collection

The outcomes included the following:

- Adherence to ENG implant use;
- Counseling effectiveness and impact on contraceptive choices;
- Adverse effects such as abnormal uterine bleeding or systemic effects.

The variables included the following:

- Participant demographics (age, BMI, etc.);
- Intervention characteristics (timing, counseling method, etc.);
- Clinical outcomes (implant retention, side effects, etc.).

Risk of Bias Assessment

Details on the tools or number of reviewers for bias assessment are not mentioned, as this was not applicable.

Effect Measures and Data Synthesis

The effect measures included the following:

- Clinical efficacy rates (Pearl Index), continuation rates, and satisfaction levels;
- Impact on adverse effects and contraceptive choice changes.

Data synthesis methods included the following:

- No meta-analysis or specific heterogeneity exploration mentioned. Results were synthesized narratively due to the nature of the study.

## 3. Results

### 3.1. Etonogestrel Subdermal Implant for Contraception: Mechanism of Action

**Figure 1:**
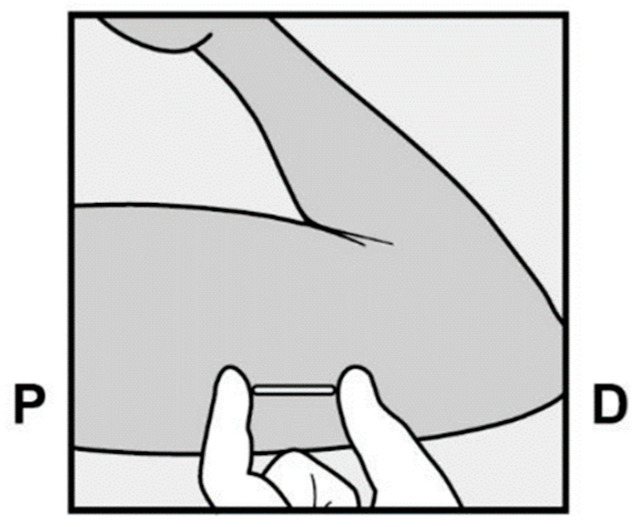
Area of placement of a subcutaneous contraceptive implant in the arm. P = proximal; D = distal.

The etonogestrel subcutaneous implant (ENG), marketed under the names Implanon and Nexplanon, is classified as a long-acting reversible contraceptive (LARC). This progestin is specifically indicated for pregnancy prevention. It is a radiopaque rod, measuring 4 cm × 2 mm, containing 68 mg of etonogestrel and pre-loaded in a single-use applicator syringe (Figure 1).

**Figure 2:**
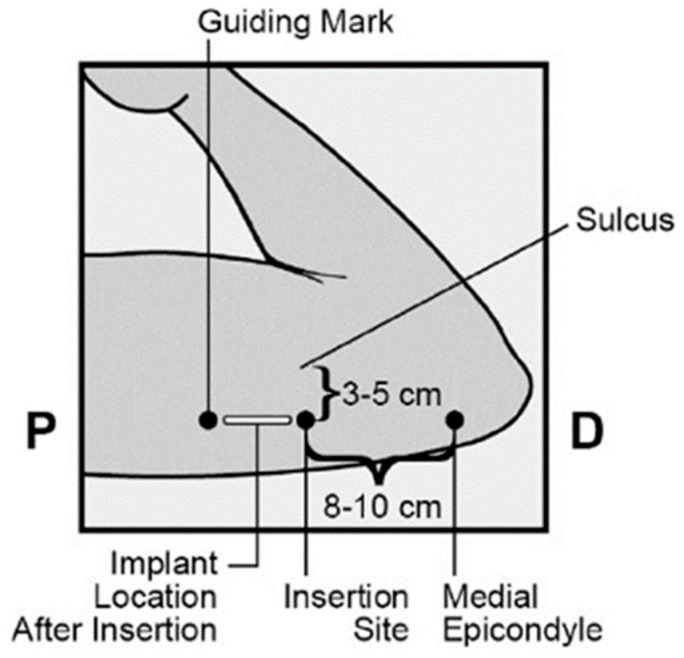
Etonogestrel implant insertion site. P = proximal (toward the shoulder); D = distal (toward the elbow).

The implant is inserted subcutaneously, specifically into the inner aspect of the non-dominant arm. Ideally, insertion should occur within the first 5 days of a typical 28-day menstrual cycle, which ensures that no additional contraceptive measures are needed. However, if insertion takes place at a different time in the cycle, it is recommended to use an additional contraceptive method for at least 7 days. The insertion site is located above the triceps muscle, approximately 8–10 cm (3–4 inches) from the medial condyle of the humerus and 3–5 cm (1.25–2 inches) posterior to the groove between the biceps and triceps muscles. This specific location is chosen to avoid the risk of damaging major blood vessels and nerves present in the groove, thus ensuring a safe and effective insertion of the implant (Figure 2) [1]. All healthcare professionals performing the insertion and/or removal of ENG must receive instruction and training before carrying out these procedures [2,3].

This contraceptive primarily works by inhibiting ovulation and inducing changes in cervical mucus and the endometrium. However, subcutaneous implants release their progestin systemically and therefore are able to suppress gonadotropin secretion and follicular maturation. Studies have reported a clinical efficacy rate of 100% and a Pearl Index ranging from 0 to 1.4 [4,5,6].

A key feature of the subcutaneous contraceptive implant, as with any long-acting reversible contraceptive (LARC) method, is its immediate reversibility and lack of impact on future fertility [2]. Etonogestrel implants are effective for 3 years, maintaining high reliability even for an additional year beyond the FDA-approved duration [8].

#### 3.1.1. Etonogestrel Subdermal Implant: Lights and Shadows

Etonogestrel implants represent a highly effective contraceptive solution for adolescents, but their acceptance is sometimes hindered by side effects.

#### 3.1.2. Abnormal Uterine Bleeding (AUB)

Changes in menstrual bleeding patterns associated with the use of the ENG implant are common. Evidence suggests a correlation between younger age, defined as adolescents aged ≤18 years, and lower BMI, typically categorized as <20 kg/m^2^, with an increased risk of AUB. For instance, studies have highlighted that adolescents in the younger age bracket and those with lower BMI are more prone to experience bothersome bleeding, which significantly impacts the continuation rates of contraceptive implants [7,9,10,11,14,15].

This phenomenon may be explained by the fact that AUB is more common with elevated serum concentrations of etonogestrel, which refer to levels above the median range observed in the general population of implant users. Studies indicate that individuals with lower BMI often have reduced adipose tissue and blood volume, potentially leading to less dilution and therefore higher circulating levels of etonogestrel. Specifically, Lazorwitz et al. [12] reported that serum concentrations of etonogestrel can vary significantly based on body composition, with lower BMI being associated with concentrations closer to or exceeding the upper quartile of observed ranges. This higher systemic exposure might account for the increased risk of AUB observed in these patients. Despite this, more than 50% of patients retained the implant up to a year after insertion [13]. In the study by Tsevat et al., 29% of adolescents and young adults developed amenorrhea after one year of using the contraceptive implant, with obese patients having twice the likelihood of experiencing it. Although no other baseline characteristics were significantly associated with amenorrhea, those who were amenorrheic at three months were more likely to remain so at 12 months [17]. Previous studies have confirmed that a high BMI is linked to fewer episodes of irregular bleeding and a higher risk of amenorrhea, likely due to the influence of endogenous estrogens on endometrial stability [16,18]. In these studies, for a BMI considered high for a 16-year-old girl, the 85th percentile corresponds to a BMI of approximately 25.2 kg/m^2^, and the 95th percentile corresponds to approximately 30.0 kg/m^2^. For a 16-year-old boy, the 85th percentile corresponds to a BMI of approximately 24.4 kg/m^2^, and the 95th percentile corresponds to approximately 28.6 kg/m^2^ [16,17,18].

#### 3.1.3. Body Weight and Body Max Index (BMI)

In a retrospective cohort study, Romano et al. examined 197 adolescents using the ENG implant over a 24-month period and found no statistically significant increases in weight or changes in BMI compared to the control group. However, 6.3% of the adolescents (*n* = 3) reported weight gain as the primary reason for early removal of the device [16].

Scott et al., analyzing the same population over a 6–18 month period, observed that adolescents with obesity experienced a threefold increase in BMI compared to those who were overweight or of normal weight [18,19]. The following examples relate BMI to age.

Considering normal weight, for a 16-year-old girl, a BMI between approximately 18.5 and 24.9 kg/m^2^ falls within the 5th to 84.9th percentiles. For a 16-year-old boy, a BMI between approximately 18.5 and 24.2 kg/m^2^ falls within the 5th to 84.9th percentiles.

Considering overweight, for a 16-year-old girl, a BMI between approximately 25 and 29.9 kg/m^2^ corresponds to the 85th to 94.9th percentiles. For a 16-year-old boy, a BMI between approximately 24.3 and 28.9 kg/m^2^ corresponds to the 85th to 94.9th percentiles.

Considering obesity, for a 16-year-old girl, a BMI of 30 kg/m^2^ or higher is at or above the 95th percentile. For a 16-year-old boy, a BMI of 29 kg/m^2^ or higher is at or above the 95th percentile.

It is important to consider that weight gain is a normal aspect of adolescent growth and development, often influenced by lifestyle changes typical of this phase, such as increased sedentary behavior and less healthy dietary habits, including a higher consumption of “fast food”, which can contribute to obesity [18,19].

#### 3.1.4. Incorrect Insertion, Breakage, and Bending of the Implant

Although rare, complications such as overly deep insertion, breakage, and bending of the implant can occur. Etonogestrel implants are radiopaque devices, which allows for their localization using X-rays. However, the risk of implant breakage increases if subjected to significant forces, such as those resulting from contact sports or collisions [20].

#### 3.1.5. Pregnancy During the Use of ENG Implants

Although ectopic pregnancies are rare, certain studies in the literature have suggested that users of the etonogestrel (ENG) implant may have a higher likelihood of experiencing an ectopic pregnancy compared to women who do not use any contraceptive method. According to the prescribing information for NEXPLANON, while the ENG implant is highly effective in preventing pregnancy, it does not eliminate the risk entirely. In cases of contraceptive failure resulting in pregnancy, ectopic pregnancy should be considered, as ectopic pregnancies have been reported in clinical settings with ENG implant users. Prompt evaluation and management are crucial for women presenting with symptoms indicative of ectopic pregnancy, such as severe abdominal pain or irregular bleeding, as untreated ectopic pregnancies can result in serious complications, including infertility or, in rare cases, mortality [21].

#### 3.1.6. Postpartum Contraception

The use of the ENG implant immediately after childbirth is considered both acceptable and effective for adolescents. Immediate insertion of the contraceptive implant offers significant economic savings by reducing the risk of closely spaced pregnancies, which is particularly relevant for this population group. Closely spaced pregnancies can lead to worse socioeconomic outcomes, poor health conditions, and complications arising from unsafe abortions [22]. Additionally, a positive association has been observed between postpartum implant insertion and significantly lower rates of postpartum depression [23,24,25,26,27].

#### 3.1.7. Pain During Contraceptive Implant Insertion

Pain perception during the insertion of the etonogestrel-based device was studied in a pilot study by Benstianov et al., which evaluated a cohort of 30 adolescent patients. The study found that the pain experienced during insertion was generally mild [28]. However, moderate levels of pre-procedure anxiety were noted [28,29].

#### 3.1.8. Systemic Hormonal Effects

The subdermal implant releases etonogestrel systemically, which can increase the risk of systemic adverse effects. Commonly reported adverse effects include allergies, breast pain, headaches, mood disturbances, and perceived worsening of chronic conditions such as nausea and dizziness. Other effects may involve intolerance to a foreign body, a desire for a break from hormones, or feeling influenced by family pressure regarding health, contraception, or sexual choices.

Additionally, damage to the implant may occur due to sports activities or trauma to the arm. Some individuals may choose to discontinue the device due to a lack of sexual activity at that time.

Approximately 14% of users reported worsening acne. However, less than 2% of users decided to stop using the implant due to this side effect [23,24].

#### 3.1.9. Comorbid Chronic Conditions

Adolescents with polycystic ovary syndrome (PCOS) may consider the ENG implant not only as a contraceptive method but also for endometrial protection. The ENG implant protects the endometrium by suppressing its proliferation, thinning the lining, and reducing estrogen receptor activity. It also alters glandular secretions and induces localized decidualization, creating an environment unsuitable for implantation. These progestogenic effects maintain an atrophic endometrium, reducing risks of hyperplasia and supporting contraceptive efficacy [2,3,4,5,6,8]. Although there are few specific studies on adolescents with chronic conditions (e.g., endometriosis, PCOS, etc.), the available data show that in the PCOS population, the continuation rate of the implant at 12 months is 77%. This result is similar to that observed among sexually active adolescents using the implant for contraception, indicating that the ENG implant is highly accepted in both populations [25].

Furthermore, a study conducted on young women with type 1 diabetes using ENG implants demonstrated a slight but significant reduction in total cholesterol, HDL, and triglyceride levels compared to T1DM adolescents using oral contraceptives [26].

## 4. Discussion and Implication for Practice

**Figure 3:**
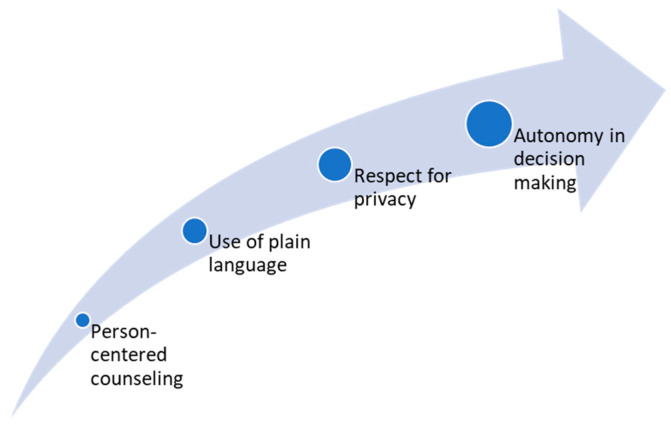
Key Elements for Effective Contraceptive Counseling.

Appropriate counseling plays a crucial role in ensuring that adolescents receive accurate and comprehensive information about available contraceptive methods, promoting informed and safe choices [22,27] (Figure 3). A well-structured approach allows young women to more accurately consider the risk of method failure while deciding which method is best suited for them. Addressing the safety and efficacy of ENG implants with adolescents and facilitating access by removing informational and economic barriers are key recommendations highlighted in the literature [30].

In the Contraceptive CHOICE Project, for example, 72% of adolescent participants chose these devices when provided with evidence-based counseling and free, easy access to all available contraceptive methods [31]. This demonstrates how appropriate counseling can positively impact adolescents’ contraceptive choices. Each visit should be seen as an opportunity to discuss reproductive life plans and, therefore, as an opportunity to reassess contraceptive use. Finally, young women who have used the etonogestrel implant for the FDA-approved duration can continue using these methods for at least an additional year without fearing a loss of efficacy.

However, there are also some barriers to the use of ENGs, as summarized in Table 1. One of the most critical aspects of contraceptive counseling for adolescents involves managing expectations and concerns regarding potential side effects. For example, abnormal uterine bleeding is one of the most common concerns associated with implant use and may discourage some adolescents from using these methods. It is essential that healthcare providers openly discuss these issues, offering evidence-based information to alleviate concerns and promote informed use of ENG implants. Additionally, it was observed that adolescents using ENG implants are 60% less likely to use condoms compared to those using oral contraceptives [32]. However, it was identified that the ENG implant does not protect against sexually transmitted infections (STIs) and HIV [33]. Therefore, this underscores the continued need for combined education encouraging the concurrent use of barrier contraceptives, such as condoms [34].

Despite the importance of effective contraceptive counseling, significant barriers persist in accessing ENG implants for adolescents. Young individuals receiving contraceptive care, whether public or private, often face unmet contraceptive needs due to the high costs associated with ENG implants, suggesting that current solutions do not adequately address the economic issue [35]. This highlights the importance of developing policies that facilitate access to safe and effective contraceptive methods regardless of income or access to financial resources.

Cognitive abilities change and mature from adolescence to young adulthood; therefore, counseling strategies must be updated to reflect these changes. It has been found that parental attitudes, especially maternal ones, are a strong determinant in their children’s contraceptive choices. It is important to consider the role, beliefs, attitudes, and involvement of parents. The literature suggests that parental involvement can significantly influence adolescents’ decisions regarding contraceptive use [30,36,37]. This emphasizes the importance of considering the role of the family in the contraceptive counseling process, with an approach that respects the adolescent’s autonomy while constructively and supportively involving the parents.

Although many adolescents arrive at counseling with a preferred contraceptive method already in mind, only a minority makes decisions completely independently of medical advice [38]. This again underscores the crucial role of healthcare providers in guiding adolescents’ contraceptive choices through open and informed dialogue that considers the specific needs and concerns of each individual.

Furthermore, adolescents may have limited knowledge about their own bodies and contraception in general. The use of visual aids and anatomical models during counseling sessions can significantly improve understanding of anatomy, physiology, and the methods of contraceptive device insertion, thereby facilitating a more informed and conscious decision-making process [39]. One study highlighted how the use of decision aids or informational videos in an obstetric–gynecological emergency room waiting area can help patients identify factors important to them in choosing a contraceptive method [40,41].

Despite the wide availability of online information, according to one study, 77% of websites do not recommend the use of ENG implants for adolescents, and 16% actively discourage their use [42]. This reflects a lack of positive recommendations and some informational resistance toward these contraceptive methods. However, recent studies showed that while messaging interventions such as text message (SMS) campaigns have increased awareness regarding sexual health, they have not had a significant impact on adherence to ENG implants among adolescents [43]. This highlights the need to explore further strategies to improve not only knowledge but also practical adherence to contraceptive methods. Additionally, while online recruitment may reduce costs and be more efficient than clinic-based strategies, it is essential to consider the nuances of social media recruitment to optimize the effectiveness of informational and counseling campaigns [44].

Healthcare providers play a fundamental role in correcting misconceptions about contraceptive methods, providing evidence-based information to promote adolescents’ autonomy [45]. They must monitor satisfaction with the chosen method, address any post-initiation concerns, and ensure the option to remove the device if necessary, to improve continuity of use and reduce early dropout rates.

Knowledge gaps among healthcare providers regarding the use of ENG implants in adolescents are common and undermine the effectiveness of counseling. In a survey of 4000 American health centers, high device costs, along with a lack of staff and specific training, were reported as significant barriers to the dissemination of these methods among adolescents [46].

Socio-cultural and economic barriers represent another obstacle to the adoption of ENGs among adolescents (Table 1). Personal acceptability of the contraceptive method is influenced by social capital, which includes experiences and social relationships that shape individual perceptions and decisions [47]. This includes misconceptions about safety and side effects, stigma associated with contraception among youth, and high device costs.

In this context, counselors need to demonstrate competence, reliability, and accessibility, addressing confidentiality issues and actively involving patients in the learning process. Kavanaugh et al. highlighted how personalized counseling that considers specific needs and economic barriers could improve the adoption and continuation of ENGs [48].

Finally, it is important to consider the specific needs of adolescents with chronic conditions, which require particularly thorough contraceptive counseling. Drug interactions and complex medical conditions must be carefully evaluated during counseling to ensure that the chosen contraceptive method is safe and effective for these patients. The vulnerability of obese adolescents to continued weight gain represents an additional risk factor that must be considered, suggesting the exploration of non-obesogenic alternatives. Informing patients at the time of implant insertion about the possibility of bothersome bleeding and available interventions to manage it could facilitate earlier follow-up and greater method acceptance.

Lastly, physicians should address potential barriers to the removal of the ENG implant before insertion. When offering the ENG implant, they can support adolescents and young women by providing flexible scheduling, such as after school, late afternoons, or weekends, and organizing such appointments both in hospitals and clinics. It is essential to inform patients about the safety and efficacy of using devices beyond FDA-approved times, if applicable, to facilitate patient-centered decisions about removal timing [37].

Additionally, gender dysphoria, a condition where a person’s gender identity does not correspond with the sex assigned at birth, can profoundly affect the well-being and quality of life of transgender adolescents. However, to date, there are no studies specifically addressing the unique challenges and barriers that transgender adolescents face in the context of contraceptive counseling. Nonetheless, it is crucial to recognize that transgender individuals, particularly transmasc adolescents, may encounter significant difficulties accessing reproductive health services, including contraception. This is often due to inadequate training or inexperience among healthcare providers, which can lead to discrimination or even denial of care. Many transmasc adolescents are at risk of unwanted pregnancies and wish to avoid menstruation, so methods like ENG implants may offer valuable options for both pregnancy prevention and menstrual cycle management. It is essential that healthcare professionals are prepared to provide tailored care that respects and affirms gender identity, ensuring equitable and safe access to contraceptive methods for all adolescents.

General Interpretation of the Results in the Context of the Living Literature

The findings of this review emphasize the high efficacy and acceptability of the etonogestrel (ENG) subdermal implant as a long-acting reversible contraceptive (LARC) for adolescents. This aligns with prior evidence highlighting LARCs as highly effective methods due to their independence from user adherence, thus reducing unintended pregnancies:

- ENG implants are shown to improve adherence and satisfaction among adolescents compared to other contraceptive methods, consistent with recommendations by ACOG and WHO;
- The barriers identified (e.g., abnormal uterine bleeding, fear of insertion) corroborate the existing literature, reinforcing the importance of tailored counseling and support to address such concerns;
- The integration of LARCs into adolescent healthcare services aligns with global public health goals, such as the UN’s 2030 Agenda for Sustainable Development.

Limitations of the evidence included in the review are as follows.

1. Population-Specific Gaps:
  - Limited studies focused exclusively on adolescents;
  - Insufficient subgroup analyses for diverse demographics (e.g., cultural or socioeconomic variations, chronic conditions, etc.).
2. Outcome Measurement:
  - Inconsistent reporting of outcomes like satisfaction, adherence, and long-term side effects;
  - Few studies examined psychosocial impacts, such as decision-making autonomy and parental influence.
3. Geographic Bias:
  - Most studies were conducted in high-income countries, potentially limiting generalizability to low- and middle-income settings where healthcare access and cultural norms may differ.

Limitations of the utilized review processes include the following.

1. Search Strategy:
  - Although broad, the search strategy may have excluded non-English literature or unpublished studies, introducing potential language or publication bias.
2. Study Selection and Screening:
  - The document does not detail whether multiple reviewers independently screened studies or resolved disagreements through consensus, which might introduce selection bias.
3. Risk of Bias Assessment:
  - No explicit tools or frameworks were used to evaluate the methodological quality of included studies, potentially undermining the robustness of the findings.
4. Synthesis Approach:
  - The narrative synthesis lacked quantitative integration, such as meta-analysis, limiting the ability to assess statistical heterogeneity or pooled effect sizes.

Implications of the results for practice, policy, and future research include the following.

1. For Practice:
  - Tailored, evidence-based counseling addressing common barriers (e.g., abnormal uterine bleeding) should be integrated into routine care for adolescents;
  - Providers should receive adequate training to deliver unbiased and comprehensive contraceptive advice;
  - Strategies to ensure equitable access, such as cost subsidies and confidential services, are essential to overcoming financial and social barriers.
2. For Policy:
  - Policies prioritizing the inclusion of LARCs in adolescent health programs can help meet public health goals, such as reducing unintended pregnancies and unsafe abortions;
  - Programs targeting underserved regions or vulnerable populations should ensure culturally sensitive and accessible contraceptive services;
  - Governments and stakeholders should address financial barriers by subsidizing LARCs or incorporating them into universal health coverage schemes.
3. For Future Research:
  - Conduct large-scale, prospective studies focusing on adolescents from diverse backgrounds to validate findings across different contexts;
  - Explore the psychosocial dimensions of LARC use, such as decision-making autonomy, stigma, and family involvement;
  - Investigate the long-term safety and efficacy of ENG implants, particularly in adolescents with chronic health conditions or obesity;
  - Develop and evaluate innovative counseling tools, including digital or visual aids, to enhance understanding and decision making among adolescents.

## 5. Conclusions

Effective contraceptive counseling is crucial for ensuring that adolescents make informed choices about their contraception. It is important for an adolescent to have open and honest discussions with their healthcare provider about the available options. Providing detailed information on the benefits and side effects of contraceptive methods such as etonogestrel implants and addressing specific concerns such as abnormal bleeding are fundamental for making an informed choice. The healthcare provider should tailor the counseling based on the adolescent’s needs and preferences, considering their medical history and lifestyle. Overcoming economic and socio-cultural barriers is essential for ensuring equitable and comprehensive access to all contraceptive methods. Thorough education and appropriate support can significantly improve adolescent reproductive health and contribute to reducing the rates of unintended pregnancies.

## Figures and Tables

**Table 1:** Barriers to the use of ENG Implants among adolescents.

Barriers to the Use of ENG Implants Among Adolescents
Knowledge gaps regarding the device
2. Inadequate training of physicians, both in counseling and proper implant placement
3. Misconceptions about safety and side effects, which may discourage adolescents from using it
4. Socio-cultural and economic context
5. Stigma associated with contraception among young adolescents
6. Costs related to device purchase
7. Menstrual flow changes, often reported as an undesirable side effect

## Data Availability

Since this is a review, no new data were generated or manipulated. The included article are publicly available in the cited databases. PRISMA flowchart and checklist available in the Supplemental Material.

## Supplementary Materials

The following supporting information can be downloaded at: https://www.mdpi.com/article/10.3390/clinpract15020027/s1. Prisma flow diagram for systematic reviews. Reference [49] is cited in the supplementary materials.
