# Supplementary figures and images for: Etonogestrel Subdermal Implant in Adolescents: Everything We Should Know to Conduct Proper Counseling, a Narrative Review
Source: Clin Pract. 2025 Jan 27;15(2):27. doi: 10.3390/clinpract15020027 (PMC11854511; doi:10.3390/clinpract15020027)

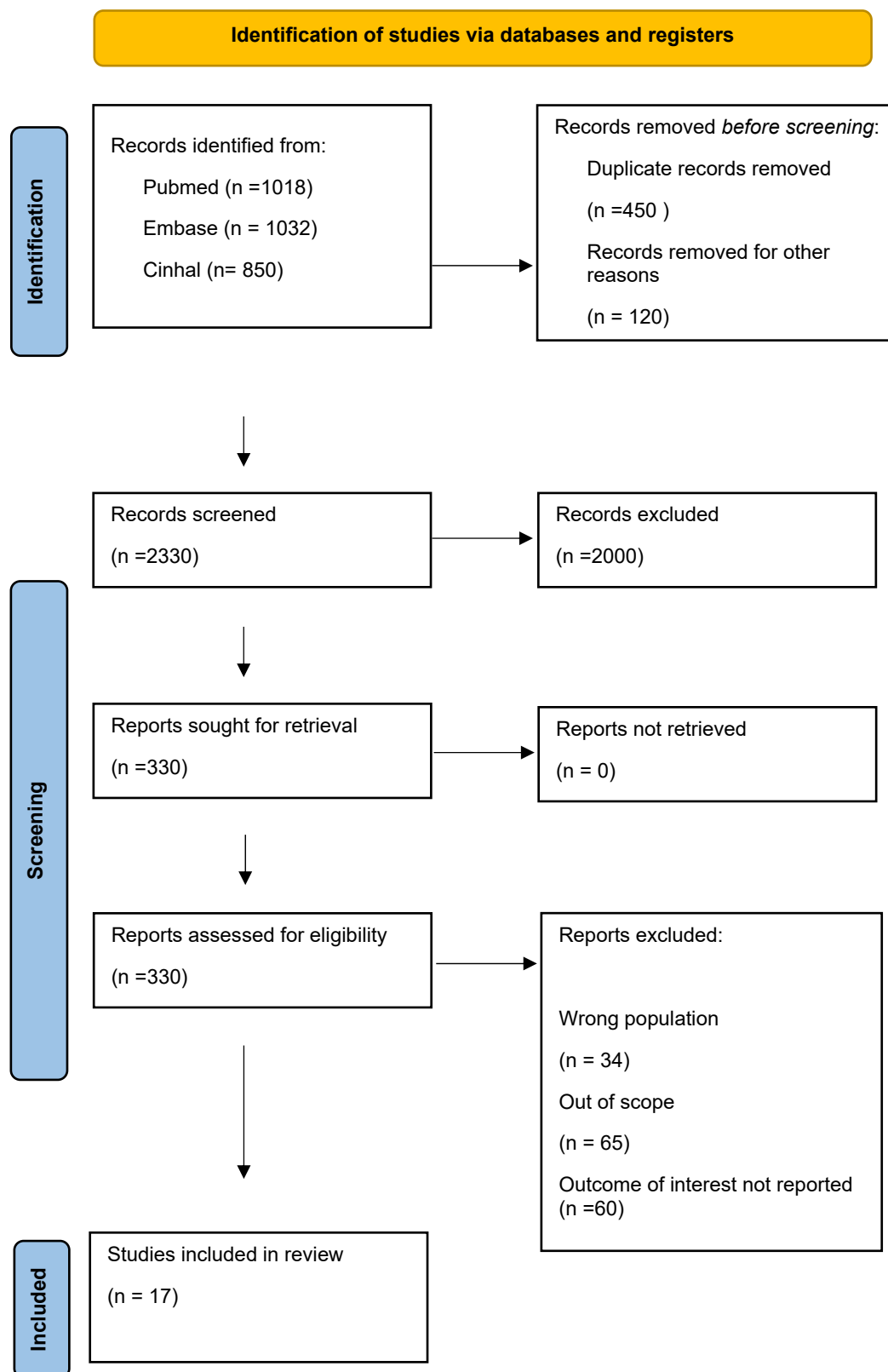

Supplement: Supplementary file 1 [file clinpract-15-00027-s001.zip › clinpract-3390574-supplementary.pdf]
